# Ru-Birds.RU, bird observations from Russia and neighbouring regions: an occurrence dataset

**DOI:** 10.3897/BDJ.9.e76202

**Published:** 2021-11-25

**Authors:** Ilya I. Ukolov, Michael S. Romanov, Vladimir Yu. Arkhipov, Mikhail V. Kalyakin, Olga V. Voltzit

**Affiliations:** 1 Russian Bird Conservation Union, Moscow, Russia Russian Bird Conservation Union Moscow Russia; 2 Institute of Mathematical Problems of Biology – branch of the Keldysh Institute of Applied Mathematics, Russian Academy of Sciences, Pushchino, Russia Institute of Mathematical Problems of Biology – branch of the Keldysh Institute of Applied Mathematics, Russian Academy of Sciences Pushchino Russia; 3 Institute of Theoretical and Experimental Biophysics, RAS, Pushchino, Russia Institute of Theoretical and Experimental Biophysics, RAS Pushchino Russia; 4 Zoological Museum of M.V. Lomonosov State University, Moscow, Russia Zoological Museum of M.V. Lomonosov State University Moscow Russia

**Keywords:** dataset, birds, Aves, occurrences, birdwatchers, GIS, Russia

## Abstract

**Background:**

The dataset covers bird observation occurrences in Russia and neighbouring regions (ex-USSR countries and some other countries of Eastern and Western Europe) from 2001–2021. It is based on the internet platform “Online bird observation diaries” (ru-birds.ru), which allows professional ornithologists and amateur bird lovers to exchange their results and to jointly build a common collection of data. The taxonomic backbone of the occurrence dataset follows the standardised GBIF checklist dataset to ensure correct cross-linking of the names.

Currently, the database contains 541,900 records of occurrences of 713 bird species, which makes it the largest dataset on birds of Russia and neighbouring regions published in GBIF.

**New information:**

The occurrence dataset contributes to filling gaps in the bird distribution in Russia and Eastern Europe. It can be used for a deeper look at their populations, phenology and migrations over this area. The availability of special tools for verification of the entered information makes the database a valuable tool for analysing occurrences of non-native species, studying vagrancy, immigration, invasions and range dynamics.

The dataset is regularly updated. Over the 11 months of 2021, it has increased by 98,165 occurrences.

## Introduction

The accuracy of the analysis largely depends on the volume and accuracy of the original information (Bar-Yam 2016). This is especially relevant for ornithology, where data on species distribution, range dynamics and migrations have always been insufficient (La Sorte et al. 2018).

In the 20^th^ century and earlier, the collection of ornithological data was the privilege of a limited number of professional researchers. During recent decades, the birdwatching movement has been gaining popularity, covering more and more countries across the world. That is why, in many countries, data collection on bird distribution, phenology, migrations and other similar work is being performed mainly by amateurs (Weisshaupt et al. 2021).

At the same time, with the development of information technology, the possibilities for collecting, exchanging and analysing information have increased many times over. In particular, online databases are being created, which allows us not only to accumulate data collected by independent observers, but also to carry out well-founded scientific analyses, based on samples from these data. These resources provide a convenient way for birdwatchers to share their materials and to become acquainted with the results of their colleagues. Obviously, the larger the number of observers, the more complete the information accumulated in a particular database. Some of these databases have grown into major international online platforms, such as eBird (Sullivan et al. 2014) or iNaturalist.

The movement of birdwatchers is also developing in Russia, but until 2013, its activity was scattered since there was no single online platform where they could interchange their results. The situation began to change with the creation of the database “Online bird observation diaries” in 2013 (Kalyakin et al. 2017). The database allows users to keep records of their observations and generate various analytical reports. It provides a convenient, intuitive interface for people with minimal knowledge of computer technology: users can enter their results either from their PC or in real-time, via mobile application.

From 2019, this online dataset was published on GBIF (Ukolov et al. 2019) as an Occurrence Darwin Core Archive (Wieczorek et al. 2012). In this article, we present the dataset itself and provide some useful information on the related online tools.

The dataset covers bird observations over a large area that is very poorly understood in terms of bird distribution compared to other parts of the world (Koblik et al. 2011). There are recent bird distribution atlases only for parts of this area or for separate bird taxa (Kalyakin and Volzit 2020, Lappo et al. 2012). Thus, there is high importance of this dataset for future works on birds' geographical distribution for this area as distribution of breeding and non-breeding ranges, as well as migration pathways, movements and occasional vagrant records.

## General description

### Purpose

The main purpose of this study is the presentation of a dataset on bird observation occurrences in Russia and neighbouring regions, published in the GBIF as a Darwin Core Archive. In addition, the study aims to provide some practically useful information on the related online database and its interface.

## Project description

### Design description

**Figure 1.**
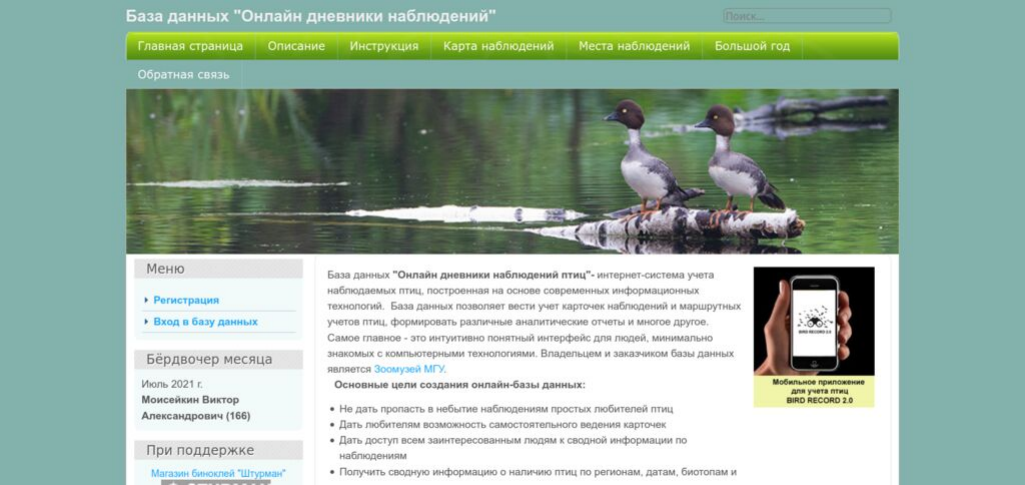
Front page of the database website RU-BIRDS.RU.

The presented dataset is a mirror of the online database “Online bird observation diaries” (Fig. 1), an Internet system for registering bird observations, created by Ilya Ukolov in 2013. The owner and customer of the database is the Moscow State University Zoological Museum (Kalyakin and Volzit 2019).

The database allows registered users to keep records of bird observations, see results of their colleagues, generate various analytical reports and much more. The main goals of creating an online database are:

- Creating an online publicly accessible database for the international scientific community;
- Keeping birdwatcher sightings from sinking into oblivion;
- Giving amateurs the opportunity to independently manage their data;
- Allowing participants to view results of their colleagues and to access summary information on the database;
- Generating summary reports on bird availability (by region, date, biotope or other features), species ranges, seasonal migrations;
- Providing an information basis for other ornithological programmes, such as preparation of the European Breeding Bird Atlas 2 (Keller et al. 2020, see also Kalyakin and Volzit 2019) and implementation of the programme “Birds of Moscow and Moscow Region” (Kalyakin and Volzit 2007, Kalyakin and Volzit 2008, Kalyakin et al. 2008).

From such a database, scientists can obtain extremely useful data on bird species distribution over time, detect species disappearing from a certain area, species invasions, migrations, occurrences of rare/endangered/non-indigenous species etc. Then they can use this information in scientific research or reports on national ecosystem services (Bukvareva and Zamolodchikov 2016).

The database was created on the Russian software “1C: Enterprise”, version 8.3 (1C Company 2015a, 1C Company 2015b), a popular environment for the development of business, home and scientific applications.

One of the important features of the database is an intuitive interface (different for PC and mobile devices), which allows users to easily upload their data and provides means for error control. The interface is provided with a map showing observation points. A user can set locations of occurrences directly on the map or manually, by entering coordinates in the text form, either by groups or one by one.

**Figure 2.**
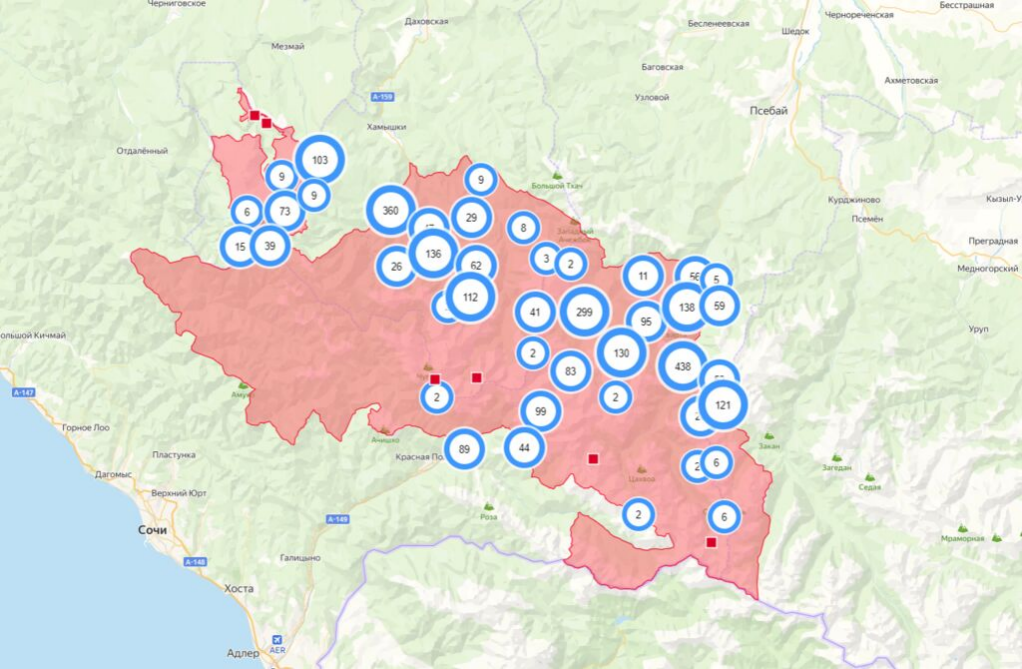
An example of a query for a manually-outlined area: the Caucasian Reserve.

It is possible to build and execute queries and produce a variety of reports. The reporting system allows users to make a selection for any period, region, species and observer and sort the output by a number of fields. Moreover, there is a possibility to manually outline the research area and obtain a query for it (Fig. 2). Along with the fully-customisable report, there are various out-of-the-box reports that users need most. The results can be saved and downloaded in several widely used formats or printed out.

Useful functionality for ornithologists, ecologists and modellers include:

- various reports;
- a map with a wide range of data display functionality;
- possibility of data analysis in the context of 10 x 10 km squares according to European Russia (with the possibility of expanding to other types of grids and to other territories);
- data analysis for an arbitrary region within arbitrary boundaries (for example, for a Reserve);
- displaying the nesting status of observed species;
- integration with GBIF, EBP.

**Figure 3.**
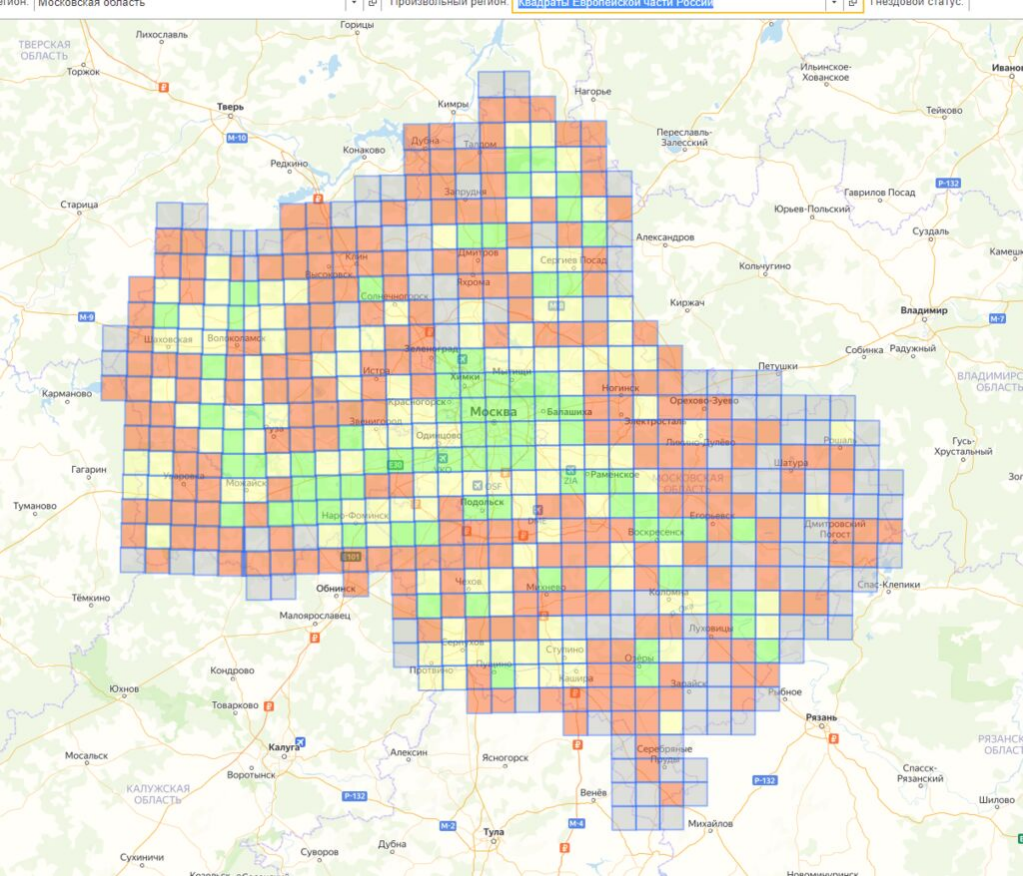
Database output in the form of squares of the European Atlas of Breeding Birds (10 × 10 km).

There are means for providing compatibility of the database with other ornithological projects. The first of them is the automatic import of data into the web GIS "Faunistics" (Karyakin et al. 2020a, Karyakin et al. 2020b). The second one is the connectivity with the squares of the pan-European Atlas of nesting birds (Fig. 3). The data from the dataset have already been used for the preparation of the European Breeding Bird Atlas 2 (Keller et al. 2020).

Being created by an amateur ornithologist, the database is well adapted for birdwatchers and possesses a number of special means related to birding activity including:

- e-mail subscription to interesting occurrences by selected region or by lifers;
- formation of regional checklists with the possibility of testing one's observation for completeness;
- a map of interesting places for observing birds (http://www.ru-birds.ru/mesta-nablyudenij.html) with automatically updated checklists;
- providing of the competition “Big Year” and rating “Club – 300” (observers who collected more than 300 species in the territory of Northern Eurasia);
- ability to control access to your own data (data closed from other users);
- displaying observation points on the map together with the species range;
- automatic identification of rare species for the region by checklist.

As an example of database analysis, let us consider the possibility of generating checklists. It is possible to generate a report for any region (both administrative and arbitrary), which presents a list of species in a given region, detailed by month. Different seasons are highlighted in colour (Fig. 7).

## Sampling methods

### Study extent

Russian Federation, 12 ex-USSR countries (Latvia, Kazakhstan, Belarus, Ukraine, Uzbekistan, Azerbaijan, Georgia, Armenia, Lithuania, Tajikistan, Estonia, Kyrgyzstan) and 12 more countries of Eastern and Western Europe (Great Britain, Belgium, Czech Republic, Luxembourg, Germany, France, Turkey, Finland, Poland, Hungary, Greece and The Netherlands).

### Sampling description

**Figure 4.**
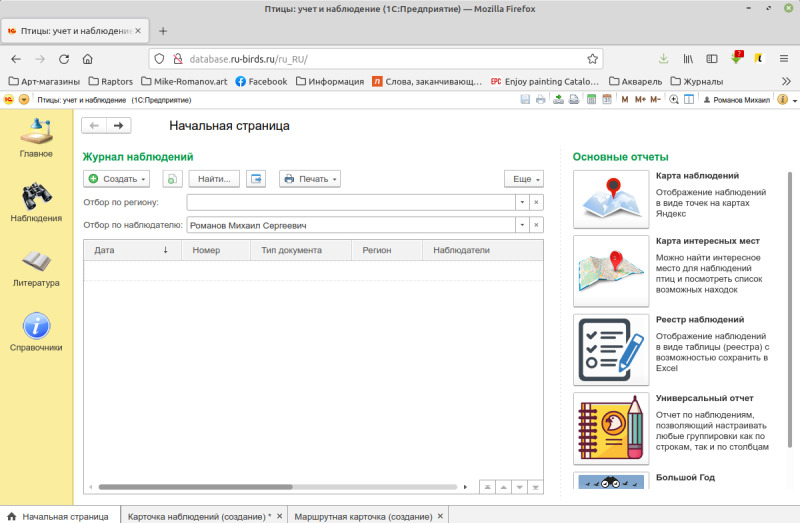
The web interface of the database.

**Figure 5.**
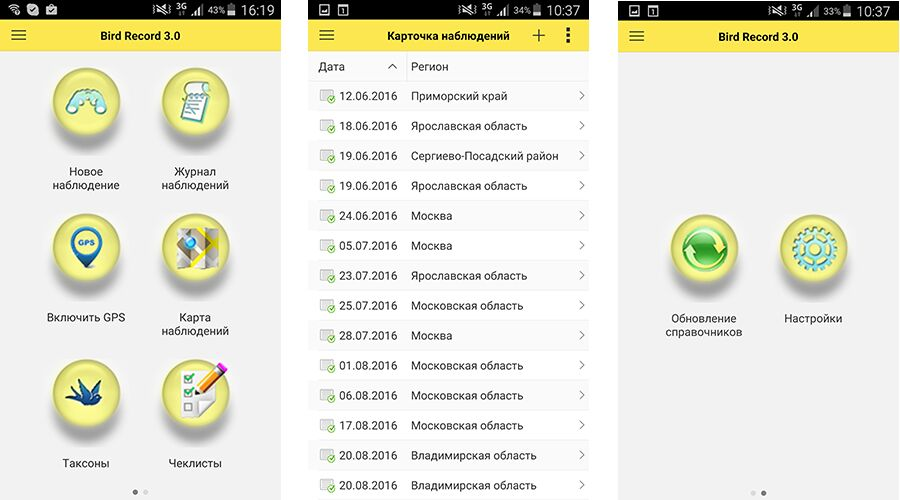
Mobile application interface.

There are two different ways of user interaction with the database, either through a browser (usually from PC; Fig. 4) or via the mobile interface (both Android and iOS applications are available; Fig. 5). Both web and mobile interfaces provide similar functionalities. For this, the web interface is best used when working with data, making queries and generating reports, while the mobile application is intended mainly for recording observations and recommended for use in the field, in real-time mode.

There are two main sampling methods: observation card and route card. The difference between them is that an observation card is essentially a checklist of observed birds together with their quantities and coordinates, while a route card is always attached to a certain route, which should be created separately. Specifically, a route card has additional fields, such as distance to the birds, the number of males and females, length of the route and time spent.

When working with an observation card, the observer enters observed species in each location together with the number of individuals. It can be done either manually or by selecting the species from the reference species list. Manual entry activates the auto-complete function, which greatly simplifies the input, allowing them to select the desired species by its first letters.

The most convenient way to indicate the location is through using a mobile application in real-time mode. In this case, the coordinates are determined by the mobile device and automatically entered in the field. When working from a PC, the user can enter the location by clicking on the digital map. Finally, the coordinates field can be filled or edited manually.

### Quality control

The database is filled both by professional ornithologists and amateurs; therefore, it is not immune to mistakes in species identification. That is why it possesses a number of means for control of data quality. Some potential errors are prevented at the stage of data entry due to the thought-out interface.

One of them is the standardised list of species (Koblik and Arkhipov 2014), which not only prevents typos, but also provides compatibility of the database with GBIF and other databases. When entering data, the programme automatically gives a hint, suggesting a list of species that can be found in the selected area. When a card is completed, it is checked for compliance with the region's checklist and, in case discrepancies are found (such as new species for the region), a warning is issued about the need for verification. Rare finds are manually checked by the database moderators separately. If needed, the identification can be corrected or, alternatively, commented in a special field of the database.

Another useful feature is the automatic determination of coordinates (by clicking on the map when working from a PC or fully automatically when using a smartphone on-the-go). Finally, some fields, such as date, can be also pre-filled automatically.

All these greatly reduce the likelihood of an error.

## Geographic coverage

### Description

**Figure 6.**
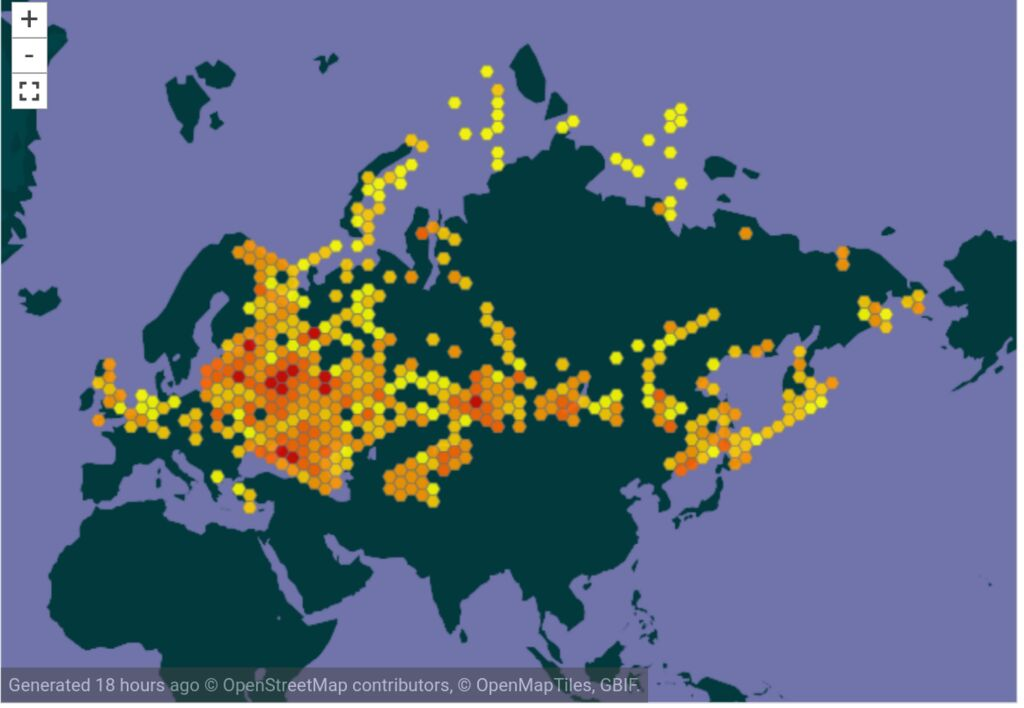
Geographic coverage of the dataset (from the dataset GBIF website).

**Figure 7.**
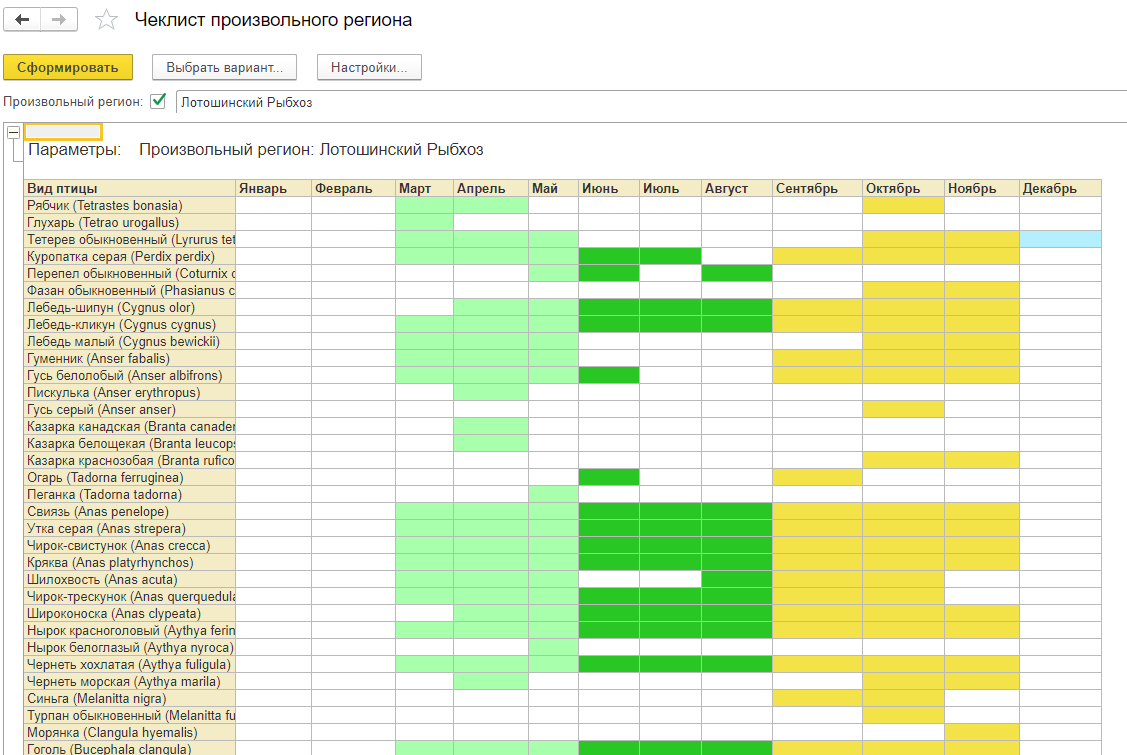
Example of database analysis: generated checklist of birds of the Moscow Region by month. Seasons are marked with different colours.

The dataset covers bird occurrences from 25 countries, including Russia, ex-USSR and some other European countries (Fig. 6). Most of the occurrences (488907 occurrences or 93%) belong to Russia. Together with five other countries, it covers 99% of the occurrences. These countries are Latvia (14401 occurrences), Kazakhstan (6568 occurrences), Belarus (4768 occurrences), Ukraine (3777 occurrences) and Great Britain (1051 occurrences). The remaining 1% of the database (4815 occurrences) is represented by 19 countries, listed in descending order: Uzbekistan, Azerbaijan, Georgia, Armenia, Belgium, Lithuania, Tajikistan, Estonia, Czech Republic, Luxembourg, Germany, Kyrgyzstan, France, Turkey, Finland, Poland, Hungary, Greece and The Netherlands.

### Coordinates

36.782 and 81.522 Latitude; 20.933 and -169.614 Longitude.

## Taxonomic coverage

### Description

The taxonomic coverage of the dataset includes 25 orders of birds, following GBIF Backbone Taxonomy (GBIF Secretariat 2021). More than 90% of occurrences belong to six orders: Passeriformes, Charadriiformes, Anseriformes, Accipitriformes, Piciformes and Columbiformes (Table 1).

### Taxa included

**Table:** 

Rank	Scientific Name	Common Name
class	Aves	Birds

## Traits coverage

### Data coverage of traits

PLEASE FILL IN TRAIT INFORMATION HERE

### Enter subsection title

Enter subsection text

## Temporal coverage

**Formation period:** 2013–2021.

## Usage licence

### Usage licence

Creative Commons Public Domain Waiver (CC-Zero)

### IP rights notes

This work is licensed under a Creative Commons Attribution Non-Commercial (CC-BY-NC) 4.0 Licence.

## Data resources

### Data package title

RU-BIRDS.RU, Birds observations database from Russia and neighbouring regions. Zoological Museum of M.V. Lomonosov Moscow State University.

### Resource link

https://www.gbif.org/dataset/ba19fc1d-670c-426b-b99d-49f003792ac4

### Alternative identifiers

https://doi.org/10.15468/5cjx70, http://www.ru-birds.ru/temp/dwca-rubirds-ru.zip, 672abba8-f26b-4256-ae27-e820258f5c8a

### Number of data sets

1

### Data set 1.

#### Data set name

RU-BIRDS.RU, Birds observations database from Russia and neighbouring regions. Zoological Museum of M.V. Lomonosov Moscow State University.

#### Data format

Darwin Core

#### Number of columns

33

#### Data format version

1.4

#### Description

Ru-Birds.RU, Birds observations from Russia and neighbouгing regions.

**Data set 1.** 

Column label	Column description
occurrenceID	An identifier for the Occurrence.
licence	A legal document giving official permission to do something with the resource.
references	A related resource that is referenced, cited or otherwise pointed to by the described resource.
datasetName	The name identifying the dataset from which the record was derived.
basisOfRecord	The specific nature of the data record.
geodeticDatum	The ellipsoid, geodetic datum or spatial reference system (SRS) upon which the geographic coordinates given in decimalLatitude and decimalLongitude are based.
recordedBy	A person, group or organisation responsible for recording the original Occurrence.
individualCount	The number of individuals present at the time of the Occurrence.
organismQuantity	A number or enumeration value for the quantity of organisms.
organismQuantityType	The type of quantification system used for the quantity of organisms.
organismName	A textual name or label assigned to an Organism instance.
eventDate	The date-time or interval during which an Event occurred. For occurrences, this is the date-time when the event was recorded. Not suitable for a time in a geological context.
year	The four-digit year in which the Event occurred, according to the Common Era Calendar.
month	The integer month in which the Event occurred.
day	The integer day of the month on which the Event occurred.
countryCode	The standard code for the country in which the Location occurs.
stateProvince	The name of the next smaller administrative region than country (state, province, canton, department, region etc.) in which the Location occurs.
decimalLatitude	The geographic latitude (in decimal degrees, using the spatial reference system given in geodeticDatum) of the geographic centre of a Location. Positive values are north of the Equator, negative values are south of it. Legal values lie between -90 and 90, inclusive.
decimalLongitude	The geographic longitude (in decimal degrees, using the spatial reference system given in geodeticDatum) of the geographic centre of a Location. Positive values are east of the Greenwich Meridian, negative values are west of it. Legal values lie between -180 and 180, inclusive.
coordinateUncertaintyInMeters	The horizontal distance (in metres) from the given decimalLatitude and decimalLongitude describing the smallest circle containing the whole of the Location. Leave the value empty if the uncertainty is unknown, cannot be estimated or is not applicable (because there are no coordinates). Zero is not a valid value for this term.
verbatimCoordinateSystem	The coordinate format for the verbatimLatitude and verbatimLongitude or the verbatimCoordinates of the Location.
identifiedBy	A list (concatenated and separated) of names of people, groups or organisations who assigned the Taxon to the subject.
scientificName	The full scientific name of the taxon (species or subspecies).
kingdom	The full scientific name of the kingdom in which the taxon is classified.
phylum	The full scientific name of the phylum or division in which the taxon is classified.
class	The full scientific name of the class in which the taxon is classified.
order	The full scientific name of the order in which the taxon is classified.
family	The full scientific name of the family in which the taxon is classified.
genus	The full scientific name of the genus in which the taxon is classified.
specificEpithet	The name of the first or species epithet of the scientificName.
infraspecificEpithet	The name of the lowest or terminal infraspecific epithet of the scientificName, excluding any rank designation.
taxonRank	The taxonomic rank of the most specific name in the scientificName.
scientificNameAuthorship	The authorship information for the scientificName, formatted according to the conventions of the applicable nomenclaturalCode.

## Additional information

It is interesting to see which of the species in the dataset are most common and which are rarest. The ten most common and the 36 rarest species are presented in Table 2 and Table 3, correspondingly, together with the number of their occurrences.

The project is continuously developing. The database is currently being integrated with EuroBirdPortal (https://eurobirdportal.org). The dataset is actively used and cited in scientific publications (e.g. Hirt et al. 2021, Oliver et al. 2021). With the growth of its volume and space-time coverage increase, the range of its possible applications also expands. We hope that, in addition to traditional tasks, such as compiling lists of species and maps of the spatial distribution of species, in the future, it will help solve a number of scientific problems, as follows:

- clarification of the boundaries of nesting and wintering areas and migration routes;
- geographical and biotopic analysis of ranges;
- phenological analysis of various events related to dispersal and migrations;
- analysis of long-term trends related to the population numbers of bird species, habitat change, the timing of migration etc.

The database fills the information vacuum concerning the number and distribution of birds that exist on the territory of Russia and neighbouring countries. Placing a dataset on GBIF and publishing its description makes the database accessible to an international circle of specialists. At the same time, this expands the potential circle of project participants.

We hope that, due to this, the volume of the database and its geographic coverage will continue to grow and, in the future, we will see new scientific papers using data from the “Online bird observation diaries” system.

## Figures and Tables

**Table 1.** Orders, presented in the dataset.

Order	No. of species	No. of occurrences
Passeriformes	332	337438
Charadriiformes	127	48960
Anseriformes	56	44142
Accipitriformes	36	26775
Piciformes	14	19699
Columbiformes	10	16711
Pelecaniformes	9	9466
Gruiformes	20	7831
Falconiformes	10	5975
Podicipediformes	5	5010
Apodiformes	4	4192
Galliformes	19	3693
Cuculiformes	5	2886
Strigiformes	17	2328
Suliformes	7	1837
Ciconiiformes	3	1633
Coraciiformes	6	1525
Bucerotiformes	1	697
Gaviiformes	5	553
Procellariiformes	10	215
Caprimulgiformes	2	173
Otidiformes	3	93
Pteroclidiformes	2	42
Phoenicopteriformes	1	16
Psittaciformes	1	9

**Table 2.** Ten most common species in the dataset.

Common name	Scientific name	No. of occurrences
Great Tit	*Parusmajor*	24919
Hooded crow	*Corvuscornix*	19076
Mallard	*Anasplatyrhynchos*	15519
Fieldfare	*Turduspilaris*	14215
Chaffinch	*Fringillacoelebs*	13462
Blue tit	*Paruscaeruleus*	11833
Black-billed Magpie	*Picapica*	11418
Rock Dove	*Columbalivia*	10791
Pied Wagtail	*Motacillaalba*	10601
Common Raven	*Corvuscorax*	10026

**Table 3.** Ten rarest species in the dataset.

Common name	Scientific name	No. of occurences
Tree Swallow	*Tachycinetabicolor*	1
Green-winged Teal	*Anascarolinensis*	1
Radde’s Accentor	*Prunellaocularis*	1
Black-headed Mountain-Finch	*Leucostictebrandti*	1
Asian Dowitcher	*Limnodromussemipalmatus*	1
Chinese Egret	*Egrettaeulophotes*	1
Gray Bunting	*Ocyrisvariabilis*	1
Aquatic Warbler	*Acrocephaluspaludicola*	1
Relict Gull	*Larusrelictus*	1
Semipalmated Plover	*Charadriussemipalmatus*	1
Pallid Scops Owl	*Otusbrucei*	1
Buller's Shearwater	*Puffinusbulleri*	1
Spotted Greenshank	*Tringaguttifer*	1
Black-faced Spoonbill	*Plataleaminor*	1
Sociable Plover	*Chettusiagregaria*	1
Least Sandpiper	*Calidrisminutilla*	1
Surf Scoter	*Melanittaperspicillata*	1
Japanese Accentor	*Prunellarubida*	1
Green-backed Heron	*Butoridesstriata*	1
Sooty Shearwater	*Puffinusgriseus*	1
Rufous-bellied Woodpecker	*Hypopicushyperythrus*	1
Chinese Pond-Heron	*Ardeolabacchus*	1
Wilson's Snipe	*Gallinagodelicata*	1
Golden-crowned Sparrow	*Zonotrichiaatricapilla*	1
Common Loon	*Gaviaimmer*	1
Great Knot	*Calidristenuirostris*	1
Streaked Shearwater	*Calonectrisleucomelas*	1
Plumbeous Water-Redstart	*Rhyacornisfuliginosa*	1
Black-headed Penduline-Tit	*Remizmacronyx*	1
Brown-headed Gull	*Larusbrunnicephalus*	1
Sombre Tit	*Paruslugubris*	1
Ivory Gull	*Pagophilaeburnea*	1
Marbled Murrelet	*Brachyramphusmarmoratus*	1
Yellow-legged Buttonquail	*Turnixtanki*	1
Pallas' Sea Eagle	*Haliaeetusleucoryphus*	1
Pechora pipit	*Anthusgustavi*	1
